# A near-complete telomere-to-telomere genome assembly for *Batrachochytrium dendrobatidis* GPL JEL423 reveals a larger CBM18 gene family and a smaller M36 metalloprotease gene family than previously recognized

**DOI:** 10.1093/g3journal/jkae304

**Published:** 2024-12-22

**Authors:** Nicolas Helmstetter, Keith Harrison, Jack Gregory, Jamie Harrison, Elizabeth Ballou, Rhys A Farrer

**Affiliations:** MRC Centre for Medical Mycology, University of Exeter, Geoffrey Pope Building, Stocker Road, Exeter EX4 4QD, UK; Biosciences, University of Exeter, Exeter EX4 4QD, UK; MRC Centre for Medical Mycology, University of Exeter, Geoffrey Pope Building, Stocker Road, Exeter EX4 4QD, UK; MRC Centre for Medical Mycology, University of Exeter, Geoffrey Pope Building, Stocker Road, Exeter EX4 4QD, UK; MRC Centre for Medical Mycology, University of Exeter, Geoffrey Pope Building, Stocker Road, Exeter EX4 4QD, UK; MRC Centre for Medical Mycology, University of Exeter, Geoffrey Pope Building, Stocker Road, Exeter EX4 4QD, UK

**Keywords:** *Batrachochytrium dendrobatidis*, genome assembly, Hi-C

## Abstract

*Batrachochytrium dendrobatidis* is responsible for mass extinctions and extirpations of amphibians, mainly driven by the Global Panzootic Lineage (*Bd*GPL). *Bd*GPL isolate JEL423 is a commonly used reference strain in studies exploring the evolution, epidemiology, and pathogenicity of chytrid pathogens. These studies have been hampered by the fragmented, erroneous, and incomplete *B. dendrobatidis* JEL423 genome assembly, which includes long stretches of ambiguous positions and poorly resolved telomeric regions. Here, we present and describe a substantially improved, near telomere-to-telomere genome assembly and gene annotation for *B. dendrobatidis* JEL423. Our new assembly is 24.5 Mb in length, ∼800 kb longer than the previously published assembly for this organism, comprising 18 nuclear scaffolds and 2 mitochondrial scaffolds and including an extra 839 kb of repetitive sequence. We discovered that the patterns of aneuploidy in *B. dendrobatidis* JEL423 have remained stable over approximately 5 years. We found that our updated assembly encodes fewer than half the number of M36 metalloprotease genes predicted in the previous assembly. In contrast, members of the crinkling and necrosis gene family were found in similar numbers to the previous assembly. We also identified a more extensive carbohydrate binding module 18 gene family than previously observed. We anticipate our findings, and the updated genome assembly will be a useful tool for further investigation of the genome evolution of the pathogenic chytrids.

## Introduction

Over 40% of amphibians are threatened with extinction (The IUCN Red List of Threatened Species 2022), attributed to several factors including pollution, habitat loss, and infectious diseases including the globally distributed chytrid fungus *Batrachochytrium dendrobatidis* (Longcore *et al*. 1999; Farrer 2019). *B. dendrobatidis* is thought to have caused the extinction of over 90 species to date, with a further 500 in decline (Scheele *et al*. 2019). *B. dendrobatidis* causes chytridiomycosis, which degrades the skin of amphibians and the keratinized mouthparts of tadpoles. Another related species called *Batrachochytrium salamandrivorans* also causes amphibian disease and biodiversity loss (Farrer 2019).

Over a decade of genomic analyses have identified genes and evolutionary processes associated with chytridiomycosis, including the expanded family of M36 metalloproteases thought to degrade host skin and extracellular matrix in both *B. dendrobatidis* and *B. salamandrivorans* (Joneson *et al*. 2011; Farrer *et al*. 2017; Wacker *et al*. 2023), the enigmatic expanded family of genes with sequence similarity to crinkling and necrosis (CRN) genes (Amaro *et al*. 2017; Farrer *et al*. 2017), as well as carbohydrate binding module 18 gene family (CBM18) thought to bind and thereby limit exposure of fungal chitin (Liu and Stajich 2015; Farrer *et al*. 2017). Several gene family expansions have been observed in the previous *B. dendrobatidis* genome assembly, including that of the M36 metalloproteases; these have been linked to transposon proliferation and evolutionary selection, especially in *B. salamandrivorans* (Wacker *et al*. 2023). Furthermore, loss of heterozygosity and whole chromosome aneuploidy have been detected in *B. dendrobatidis* genomes, possibly linked to its pathogenic lifestyle (Farrer *et al*. 2013). However, the specific genes and mechanisms underlying *B. dendrobatidis* pathogenicity remain poorly understood, owing to a lack of experimental validation of these pathogenicity factors and dated genomic resources.

*B. dendrobatidis* has genetically diverse populations, comprising 5 known lineages to date: the Global Panzootic Lineage (*Bd*GPL), *Bd*ASIA-1, *Bd*ASIA-2, *Bd*ASIA-3, and *Bd*CAPE (Farrer *et al*. 2011; O’Hanlon *et al*. 2018; Wacker *et al*. 2024). Of the 5 known lineages, *Bd*GPL is characterized as hypervirulent and globally distributed (Fisher and Garner 2020) and is thought to be the main driver of the chytridiomycosis panzootic (Farrer *et al*. 2011). *Bd*GPL isolate JEL423 was originally isolated by Dr. Joyce Longcore at the University of Maine from a lemur leaf frog (*Agalychnis lemur*) in Panama. *B. dendrobatidis* JEL423 was sequenced and assembled in 2006 into 69 scaffolds with a total length of 23.7 Mb, using a hybrid approach including Sanger and Illumina data [no manuscript accompanied this data, although the revised annotation and polished assembly is described here (Farrer *et al*. 2017)].

In 2017, the assembly was updated and improved (version 2 of the assembly) using Illumina DNAseq-based Pilon polishing and gene annotation refinement using RNA-seq (Farrer *et al*. 2017). In 2018, the structure of the mitochondrial genome was resolved using long-read sequencing, into 3 linear segments (O’Hanlon *et al*. 2018). Here, we describe an updated, near telomere-to-telomere genome assembly and gene annotation for *B. dendrobatidis* JEL423 (version 3) that will be a crucial resource for understanding *B. dendrobatidis*' evolution to a pathogenic lifestyle.

## Materials and methods

### DNA extraction and sequencing

*B. dendrobatidis* strain JEL423 zoosporangia and zoospores were cultured in half strength tryptone gelatin hydrolysate lactose (TGhL) (0.8% tryptone, 0.1% gelatin hydrolysate, and 0.2% lactose) broth in static cell culture flasks at 18°C. High molecular weight DNA was obtained by a customized cetyltrimethylammonium bromide (CTAB) extraction procedure (Schwessinger 2019 May 17). Briefly, cell pellet was ground with a mortar and pestle in liquid nitrogen, incubated in a CTAB lysis buffer with proteinase K (460 units/mL) and RNAse A (570 units/mL), followed by potassium acetate precipitation, phenol chloroform isoamyl alcohol purification, and isopropanol precipitation. Extracted DNA was checked for integrity on agarose gel and for purity and concentration with NanoDrop and Qubit. After the first extraction, 260/230 absorbance was 1.62 and the ratios between concentrations measured with Qubit and NanoDrop was 1/8. DNA was further purified with a QIAgen Plant Pro column resulting in 260/230 absorbance of 2.82 and concentrations ratio of 1.

An ONT library was prepared by the Exeter Sequencing Service with kit SQK-LSK109 (Oxford Nanopore Technologies) following the manufacturer's instructions. Briefly, DNA ends were FFPE repaired and end-prepped/dA-tailed using the NEBNext FFPE DNA Repair kit (M6630, NEB) and the NEBNext Ultra II End-Repair/dA-tailing Module (E7546, NEB) followed by AMPure XP bead clean-up (A63882, Beckman Coulter). Adapters were ligated using the Genomic DNA by Ligation kit (SQK-LSK109, Oxford Nanopore Technologies) and NEBNext Quick T4 DNA Ligase (E6056, NEB) followed by AMPure XP bead clean-up. The library was loaded on a Flongle flow cell (FLO-FLG001). Sequencing run yielded 1.1 Gb with N_50_ of 19.4 kb. Basecalling was achieved using dorado v0.5.0 (0.5.0 + 0d932c0) (https://github.com/nanoporetech/dorado) with the arguments “–recursive –device='cuda:1' –emit-fastq dna_r9.4.1_e8_sup@v3.6 ./fast5”.

A Hi-C library was constructed using an Arima High Coverage Kit (Arima Genomics, CA, USA) according to the manufacturer's instructions for mammalian cell lines (A160161 v01) and the Arima Library Prep Module (A160186 v01). Briefly, synchronized *B. dendrobatidis* cultures were filtered with a 10 µm cell strainer (pluriselect) to isolate zoospores, followed by in situ cell crosslinking, cell lysis chromatin digestion with 4 restriction enzymes (^GATC, G^ANTC, C^TNAG, T^TAA), biotin labeling, proximal chromatin DNA ligation, and DNA purification. The resulting DNA was fragmented by sonication (Covaris E220) and enriched in biotinylated fragments followed by library preparation. The Hi-C library was subjected to paired-end sequencing with 150 bp read lengths on an Illumina NovaSeq platform, resulting in 166 million read pairs (49.8 Gb, 2011× coverage).

### Genome assembly

The LongHam pipeline (long-read hybrid assembly merger) (Marcet-Houben *et al*. 2022) was used to assemble ONT reads for *B. dendrobatidis* JEL423 (this study) and paired Illumina reads for *B. dendrobatidis* JEL423 (Farrer *et al*. 2013) to generate candidate primary assemblies. The hybrid de novo genome assembly that demonstrated the best mix of contiguity, completeness, and number of telomeres was generated by the Maryland Super-Read Celera Assembler v.4.1.0 (MaSuRCA) (Zimin *et al*. 2013) with parameters “GRAPH_KMER_SIZE = auto, USE_LINKING_MATES = 0, LIMIT_JUMP_COVERAGE = 300, CA_PARAMETERS = cgwErrorRate = 0.15, KMER_COUNT_THRESHOLD = 1, NUM_THREADS = 16, JF_SIZE = 480000000, SOAP_ASSEMBLY = 0”. For polishing, Pilon v1.24 (Walker *et al*. 2014) was used with Illumina paired-end reads aligned to the draft assembly and indexed using BWA v.0.7.17 (Li 2013 Mar 16). The draft assembly and the corresponding alignments were then passed to Pilon v.1.24 (Walker *et al*. 2014) to call the consensus sequence.

Hi-C reads were aligned to the de novo assembly following the Arima mapping pipeline (https://github.com/ArimaGenomics/mapping_pipeline). Paired-end Illumina reads were first mapped independently to the genome sequence using BWA mem (Li 2013 Mar 16). Next, chimeric reads were filtered to only retain the 5′ side. Reads were then sorted, mapping quality filtered and paired, followed by removal of PCR duplicates. The final output is a single BAM file that contains the paired, 5′-filtered, and duplicate-removed Hi-C reads, mapped to the reference sequence. Contigs were scaffolded with YaHS version 1.1 (Zhou *et al*. 2023). Mis-assemblies of contigs were manually corrected based using the resulting Hi-C contact map with Juicer Tools version 1.19.2 (Dudchenko *et al*. 2018) and Juicebox version 2.15 (Dudchenko *et al*. 2018). The resulting assembly has 18 scaffolds and 11 gaps. TGS-GapCloser version 1.1.1 (Xu *et al*. 2020) was used to fill gaps in the scaffolded assembly [using the long reads corrected with the short reads by Pilon v1.24 (Walker *et al*. 2014)]. A total of 7 gaps were closed resulting in the final assembly of 18 scaffolds and 4 gaps. Scaffold ends were screened for telomeric sequences (TTAGGGn) with find_telomeric_repeats.py that is part of the LongHam pipeline (Marcet-Houben *et al*. 2022).

To calculate mapping rate, we aligned Illumina reads to the genome assembly using BWA v0.7.17 (Li 2013 Mar 16) and ONT long reads using minimap2 v2.28 (Li 2018). The rate was then calculated with SAMtools v1.20 flagstat (Li *et al*. 2009). To assess assembly completeness, we searched the *B. dendrobatidis* JEL423 genome sequence for a panel of core genes using Benchmarking Universal Single-Copy Orthologs (BUSCO) version 5.7.0 (Manni *et al*. 2021) specific to the fungi odb10 database.

### Genome annotation

The genome assembly was screened for repetitive sequence and masked using RepeatModeler2 (Flynn *et al*. 2020) and RepeatMasker version 4.1.5 (Smit et al.). Braker3 (Gabriel *et al*. 2024) was used to annotate the genome with esmode (for gene prediction with genome sequence data only) and softmasking enabled which combines GeneMark-ET version 3.68 (Lukashin and Borodovsky 1998) and AUGUSTUS version 3.5.0 (Stanke *et al*. 2006). EDTA version 2.2.0 (Ou *et al*. 2019) was used to annotate and classify transposable elements across the genome. We predicted the presence of signal peptides using SignalP4 (Petersen *et al*. 2011).

The protease composition of each chytrid was determined using top high scoring pairs from BLASTp searches (e-value < 1e-5) made to the file “pepunit.lib”, which is a nonredundant library of protein sequences of all the peptidases and peptidase inhibitors that are included in the MEROPS database (release 12.4) (Rawlings *et al*. 2016). All proteases with matches to M36 metalloproteases were aligned using MUSCLE v5.1.osx64 (Edgar 2022). We constructed the gene trees with FastTree version 2.1.11 SSE3 (Price *et al*. 2009) with default parameters and visualized with FigTree v1.4.4 (http://tree.bio.ed.ac.uk/software/figtree/).

Crinklers and CBM18s were predicted based on BLASTp searches (e-value < 1e-5) to previously identified sequences (Farrer *et al*. 2017). Two additional putative CBM18s (g4693 and g4694) were identified by HMMER v.3.1b2 (Eddy 2011) (hmmbuild the PFAM PF00187 and hmmscan all proteins). Interpro domain searches were used to identify and name domains. Completion of M36 metalloprotease and crinkler gene annotation was checked using tblastn v.2.15.0+ against our new *B. dendrobatidis* JEL423 genome assembly, and top HSPs and any overlapping predicted features were manually checked. Putative crinkler protein sequences were aligned using MUSCLE v5.1.osx64 (Edgar 2022) and gene trees constructed with FastTree version 2.1.11 SSE3 (Price *et al*. 2009).

### Orthology prediction, synteny, aneuploidy, and phylogenetics

Orthologs were predicted between our new *B. dendrobatidis* JEL423 annotation and the previous (V2) assembly and annotation using the Synima pipeline (Farrer 2017) with OrthoMCL (Li *et al*. 2003). We also performed comparative genomics between *B. dendrobatidis* and 6 of its closest relatives (Wacker *et al*. 2023) that were downloaded from the MycoCosm portal of the US Department of Energy (DOE) Joint Genome Institute (JGI) (Grigoriev *et al*. 2014) (specifically, *Gorgonomyces haynaldii* MP57 (Amses *et al*. 2022), *Globomyces pollinis-pini* Arg68 (Amses *et al*. 2022), *Homolaphlyctis polyrhiza* JEL142 (Farrer 2016), *B. salamandrivorans* AMFP13 (GCA_002006685.2) (Wacker *et al*. 2023), and *Entophlyctis helioformis* JEL805 (Amses *et al*. 2022). Additionally, we included the genome for recently discovered *Polyrhizophydium stewartii* JEL0888 (GCA_027604665.1) (Simmons *et al*. 2021). The Synima pipeline outputs Orthogroups, which we divided into categories of interest, including single copy orthologs, which we used to construct phylogenetic trees. We generated a phylogenetic tree using 1,186 single copy orthologs. Single copy orthologs were aligned individually using MUSCLE version 5.1.osx64 (Edgar 2004) with default settings. We concatenated all alignments into a single FASTA and converted that into nexus format for phylogenetic analysis. We used the PROTCATWAG model for protein evolution with RAxML v.8.2.12 (Stamatakis 2014) with 1,000 bootstraps. Plotting ploidy and allele frequencies were performed using bespoke Perl scripts processing the data in pileup format (https://github.com/rhysf/allele_frequencies).

## Results

**Fig. 1.**
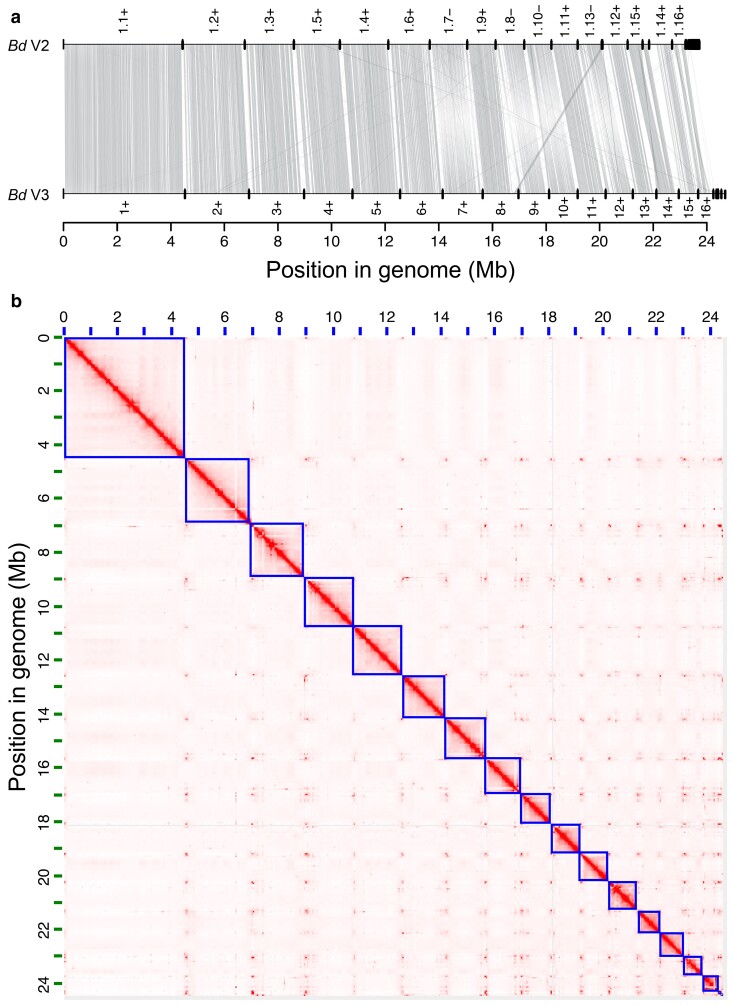
a) Synteny plot of previous assembly and annotation for *B. dendrobatidis* JEL423 (V2) compared with the new version (V3). Scaffold numbers are shown, along with the orientation. b) A chromatin capture contact map of *B. dendrobatidis*, where squares indicate putative chromosomes and bolder red indicates increased contact frequency. The Rabl-like phenomenon found in other species, where telomeres co-situate in the nucleus, is visible.

We reassembled *B. dendrobatidis* JEL423 using long Oxford Nanopore Technologies (ONT) and Hi-C sequencing, which substantially improved assembly metrics compared with the previous V2 assembly (Fig. 1; Table 1). Specifically, the V2 23.7 Mb assembly is highly fragmented (69 scaffolds, 348 contigs) and includes many long stretches of ambiguous bases (318,766 Ns in total), while our new assembly is ∼800 kb longer (24.5 Mb), comprising just 18 nuclear scaffolds, 2 mitochondrial scaffolds, 22 contigs, and only 4 gaps. The final Hi-C contact map (Fig. 1b) validates the contiguity of the assembly, showing the strong self-interactions of scaffolds 1 to 16 and the clear junctions between each of them, typical of chromosomes. Those chromosomes seem to be organized in a Rabl-like architecture with prominent contacts between telomeres (Hoencamp *et al*. 2021).

**Table 1.** Genome assembly metrics of the previous *B. dendrobatidis* assembly (V2) compared with our updated genome assembly (V3).

Genome assembly	JEL423 (V2)	JEL423 (V3)
Total length (Mb)	23.7	24.5
Contig count	348	22
Contig N_50_ (Mb)	0.22	1.49
Contig N_90_ (Kb)	42	752
Contig L_50_	32	6
Contig L_90_	129	15
Scaffold count	69	18
Scaffold N_50_ (Mb)	1.70	1.77
Scaffold N_90_ (Kb)	857	877
Scaffold L_50_	5	5
Scaffold L_90_	14	13
Number of gaps	279	4
Ns per 100 kb	13.44	0.02
Largest scaffold (Mb)	4.4	4.5
Telomeres total	0	18
Telomeres forward (5′)	0	8
Telomeres reverse (3′)	0	10
Telomere-to-telomere scaffolds	0	5
GC content (%)	39.29	39.28
Protein coding genes	8630	8628
BUSCO complete (%)	91.4	92.1
Short-read alignment rate (%)	94.36	99.03
Long-read alignment rate (%)	93.27	96.17

To verify the accuracy of the improved genome assembly, we calculated mapping rates by aligning Illumina data (O’Hanlon *et al*. 2018) and long reads (this study) to the genome assembly. As a result, 99.02% of the Illumina reads aligned (up from 94.36% for *B. dendrobatidis* V2) and 96.17% of the long reads aligned (up from 93.27% for *B. dendrobatidis* V2). Furthermore, the V2 assembly had no contigs ending in telomeric repeats, while our updated assembly is near telomere-to-telomere, with 13/18 (72%) scaffolds terminating with 1 or 2 telomeric repeat (TTAGGG), while 5/18 (28%) have telomeres at both ends (Supplementary Table 1). Finally, our new, more contiguous mitochondrial genome assembly is ∼ 260 kb: a reduction from the previous assembly of nearly 300 kb.

**Fig. 2.**
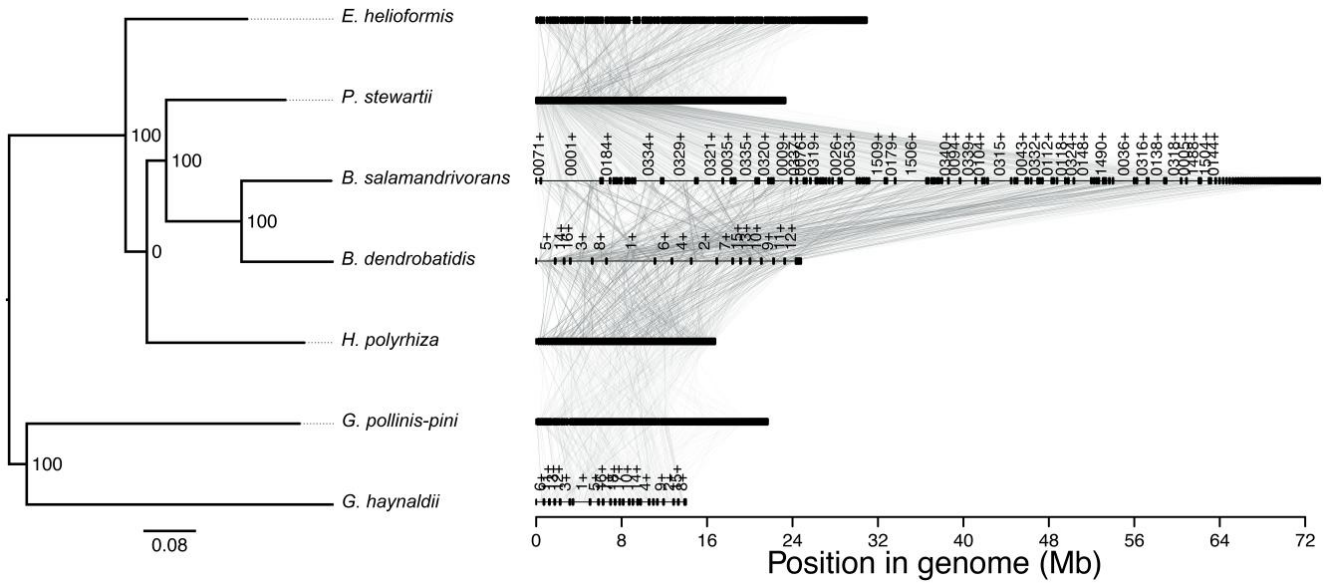
A synteny plot between the new *B. dendrobatidis* genome assembly and its closest relatives. Synteny is poorly conserved between the species represented. Additionally, the genome assemblies of all species apart from our new *B. dendrobatidis* JEL423 assembly reported here remain fragmented or highly fragmented.

We reannotated our assembly, which identified 8,628 predicted protein coding genes (excluding splice variants), which was very similar to previously predicted (*n* = 8,630). BUSCO scores from the new assembly were slightly improved compared with the V2 assembly (92.1% up from 91.4%), suggesting a higher overall accuracy in assembly and gene prediction. Our improved genome assembly demonstrated many genomic rearrangements compared to the previous assembly, in addition to the increased overall length and contiguity (Fig. 1a). Comparing the new *B. dendrobatidis* assembly to genome assemblies from its closest-known chytrid relatives supported the relationships and the low level of conserved synteny previously identified between those species, as well as highlighting the fragmented and low-quality assemblies of those other species being compared to (Fig. 2).

Our new assembly facilitated the prediction of a further 839 kb of repetitive sequence compared with the previous assembly (23.89% instead of 21.42%; Table 2). Notably, increases in the numbers of unclassified and total interspersed repeats, long terminal repeat (LTR) elements, DNA transposons, and retroelements, as well as small RNAs, were identified in the new assembly. Mutator, Cacta, and Maverick-type DNA transposons are the most common in the *B. dendrobatidis* genome assembly followed closely by retrotransposons (Supplementary Table 2). Conversely, there were small decreases in the numbers of short interspersed nuclear elements, long interspersed nuclear elements, and low complexity repeats.

**Table 2.** Repeat content predicted from the previous *B. dendrobatidis* assembly (V2) compared with our updated genome assembly (V3) based on RepeatMasker and RepeatModeler.

	JEL423 (V2)			JEL423 (V3)			
	Number	Length (bp)	Genome occupied (%)	Number	Length (bp)	Genome occupied (%)	Difference (nt)
Total (bases masked)		5,013,710	21.42		5,852,638	23.89	838,928
Retroelements	265	267,526	1.14	513	297,044	1.21	29,518
SINEs	45	30,456	0.13	28	17,579	0.07	−12,877
LINEs	108	152,775	0.65	62	129,726	0.53	−23,049
LINEs (R1/LOA/Jockey)	19	12,358	0.05	0	0	0	−12,358
LTR elements	112	84,295	0.36	423	149,739	0.61	65,444
LTR elements (BEL/Pao)	0	0	0	322	52,204	0.21	52,204
LTR elements (Ty1/Copia)	63	63,588	0.27	75	86,851	0.35	23,263
LTR elements (Gypsy/DIRS1)	33	10,112	0.04	26	10,684	0.04	572
LTR elements (Gypsy/DIRS1—retroviral)	16	10595	0.05	0	0	0	−10,595
DNA transposons	565	775,071	3.31	719	804,803	3.28	29,732
Unclassified	9,174	3,910,035	16.71	9,409	4,448,145	18.15	538,110
Total interspersed repeats		4,952,632	21.16		5,549,992	22.65	597,360
Small RNA	0	0	0	50	234,327	0.96	234,327
Simple repeats	1,191	54,799	0.23	1,310	63,418	0.26	8,619
Low complexity	115	6,279	0.03	99	4,901	0.02	−1,378

**Fig. 3.**
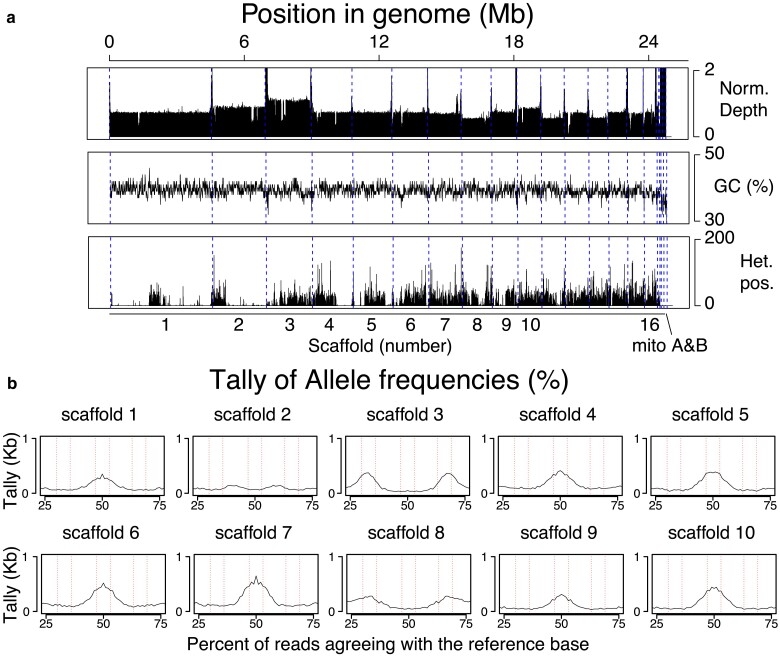
Ploidy of *B. dendrobatidis* JEL423 was assessed, based on a) normalized depth of coverage across 10 kb nonoverlapping windows, GC content, and tallying of heterozygous positions across the genomes and b) allele frequencies, where the percent of reads agreeing with the reference base was counted and tallied across every base in each chromosome, and the percents between 25 and 75% plotted. These analyses support previous findings that *B. dendrobatidis* JEL423 has widespread aneuploidy, including diploidy, triploidy, and perhaps tetraploidy+. Furthermore, loss of heterozygosity is seen across the genome, particularly in scaffolds 1, 2, and 4.

The ploidy of *B. dendrobatidis* JEL423 was assessed using previously generated Illumina reads (O’Hanlon *et al*. 2018) for the isolate aligned to the new assembly, revealing widespread aneuploidy and high base ploidy level for this isolate as previously described (Farrer *et al*. 2013) (Fig. 3). For example, normalized depth showed differences between scaffolds, with scaffolds 2, 3, 8, 10, 11, 13, 15, and 16 all at different levels to scaffold 1, and about 4 separate depth levels identified (Fig. 3a). There were also dips in normalized depth at various points within scaffolds 1, 2, 3, 4, 6, and 10 suggestive of centromeric regions. While GC content is relatively stable across the genome, loss of heterozygosity is stark, with several genomic regions showing an enrichment or dearth of heterozygosity. For example, the middle of scaffold 1, the start of scaffold 2, and half of scaffold 4 have high levels of heterozygosity, while the majority of scaffold 1 and ends of scaffolds 2 and 4 are particularly lacking in heterozygosity (Fig. 3a).

We counted the percent of reads agreeing with the reference base across every scaffold to help predict ploidy (Fig. 3b). Most positions had ∼100% of reads agreeing (reference/homozygous positions), and a smaller number had ∼0% of reads agreeing (SNP/homozygous or assembly errors) as expected, neither of which are shown in Fig. 3b. Many genomic positions indicative of heterozygosity were also identified (Fig. 3b). For example, scaffold 1 has a biallelic peak suggestive of an even ploidy (such as diploid or tetraploid), scaffold 2 has slightly higher depth and ambiguous or little evidence of heterozygosity (owing in part to the loss of heterozygosity for most of that scaffold), and scaffold 3 has higher depth than scaffolds 1 and 2 and has peaks at 33 and 67% indicative of an odd ploidy of triploid or higher, while scaffold 4 returns to scaffold 1 depth of coverage and has a biallelic peak suggestive of an even ploidy. Scaffold 8 (along with scaffolds 11 and 13) has the lowest normalized depth level (Fig. 3a), and scaffold 8 shows trimodal heterozygosity peaks suggesting triploid, which together with the above results, suggest *B. dendrobatidis* JEL423 is base tetraploid, with some triploid and pentaploid scaffolds. More surprising is that the pattern of normalized depth and allele frequencies is very consistent with the previous assemblies and the depth of coverage and allele frequencies identified from our 2013 paper (Farrer *et al*. 2013) and 2018 paper (O’Hanlon *et al*. 2018), with sequencing (and culturing) occurring approximately 5 years apart, indicating that this aneuploidy is not transient as previously speculated (Farrer *et al*. 2013), but may be stable, at least across several years in passage and/or cryogenically frozen stocks.

**Fig. 4.**
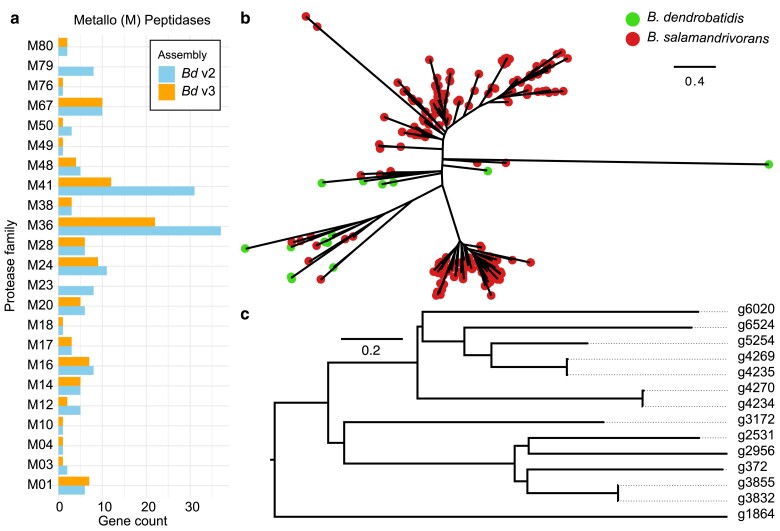
Predicted M36 metalloproteases in *B. dendrobatidis* JEL423. a) Metalloproteases had a modest difference in total count between the old (V2) and new (V3) assemblies, with substantial decreases in the number of M36 and M41 proteases. b) A gene tree (FastTree) between *B. dendrobatidis* JEL423 (V3) and *B. salamandrivorans* M36's supports previous findings of multiple subgene families and ancestral gene expansions, particularly in *B. salamandrivorans* since its split with *B. dendrobatidis*. c) A gene tree (FastTree) of predicted *B. dendrobatidis* M36's. Branch lengths indicate the mean number of nucleotide substitutions per site.

We found a reduction in the number of encoded M36 metalloprotease, which are a key predicted pathogenicity factor, in the new assembly (*n* = 14) compared with the old assembly (*n* = 37) (Fig. 4). Indeed, we found fewer predicted proteases overall (*n* = 722) compared with the previous assembly (*n* = 804) (Supplementary Fig. 1). To ensure the reduction in number of M36 metalloprotease genes was not a result of erroneous gene calling, we used tblastn of all our new M36 genes to the genome assembly, identifying 8 further genes with sequence similarity (Supplementary Fig. 2). However, only one of these newly identified genes had predicted protease function (A1 protease g2465). The majority of the newly identified M36 genes belonged to an orthogroup with M36 in the V2 assembly (*n* = 10/14). The newly identified M36 genes fall within the *B. dendrobatidis* and *B. salamandrivorans* metalloprotease clade as previously identified (Fig. 4, b and c). However, the reduced number in *B. dendrobatidis* suggests that the gene expansion of M36s in *B. salamandrivorans* is even more substantial than previously recognized.

Unlike the M36 metalloproteases, a large expansion of CRN genes is still predicted as a unique feature of the *B. dendrobatidis* genome. Using BLASTp for all proteins against the CRNs predicted in *Phytophthora infestans* T30-4 (where CRNs are better described) as previously performed (Farrer *et al*. 2017) revealed only 13 top high scoring pairs (HSPs) after excluding splice variants (Supplementary Fig. 3). However, after running tblastn against the genome, 133 separate putative CRNs were identified, which is similar to the number previously identified in the V2 assembly (*n* = 162; 82%) (Supplementary Fig. 4).

**Fig. 5.**
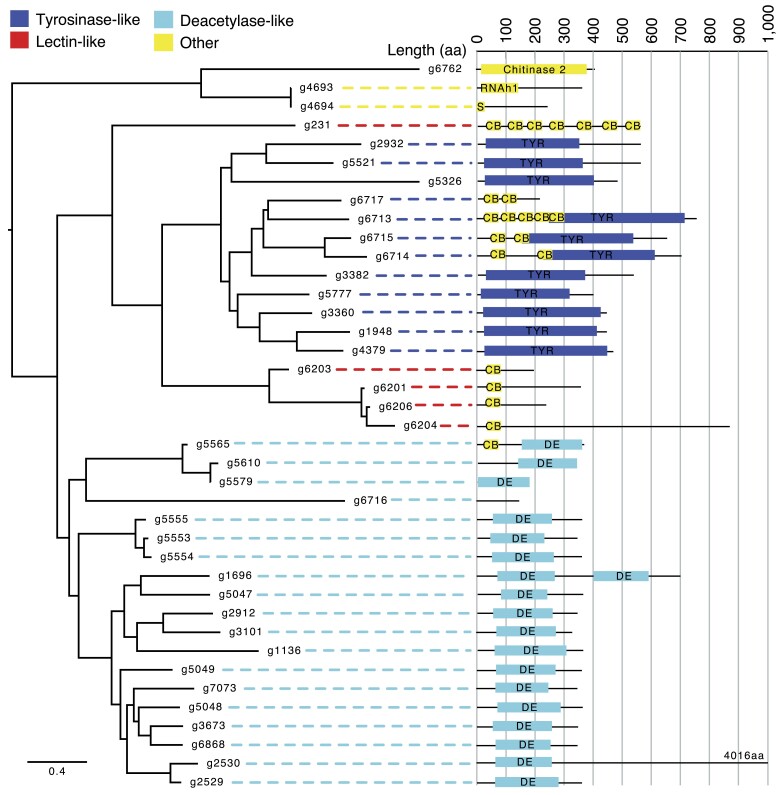
Predicted CBM18 genes in *B. dendrobatidis* JEL423. A phylogenetic tree of all CBM18 proteins based on BLAST to previously identified CBM18s and HMMER to PF00187, aligned using MUSCLE and a tree constructed with FastTree (branch lengths indicate the mean number of nucleotide substitutions per site). To the right of gene names is a diagram of the domain structure (RNAh1, RNase H type-1; S, secretion signal; CB, carbohydrate binding; TYR, tyrosinase copper-binding domain; DE, glycoside hydrolase/deacetylase). Lectin-like are based on sequence similarity (BLASTp) to previously identified proteins (Liu and Stajich 2015).

The CBM18 gene family has nearly double the predicted genes in the updated assembly compared with the V2 assembly (*n* = 39 compared with 22) (Fig. 5). Based on BLASTp and HMMER searches, the number of tyrosine-like increased from 5 to 12, the number of deacetylase-like increased from 10 to 19, and the number of lectin-like remained at 5. While the tyrosine-like and deacetylase-like were easy to predict based on PFAM domains, the lectin-like were less clear, as these were based on previous predictions, and the BLASTp searches overlapped with deacetylase-like genes. Notably, gene g231 has the lowest BLASTp e-value (e-value = 0, compared with the next lowest at 1.09e-51 for g6713 which is clustered with and contains a tyrosine-like PFAM domain).

## Discussion

*B. dendrobatidis* is responsible for mass extinctions and extirpations of amphibians. *B. dendrobatidis* isolate JEL423 was initially isolated from a lemur leaf frog in Panama during a notable extinction event across Central America, and the genome was assembled in 2006 (Farrer *et al*. 2017). Since then, the reference genome of *B. dendrobatidis* JEL423 has been widely used to help understand, track, and predict the species evolution, epidemiology, and pathogenicity (Farrer *et al*. 2011, 2017; Joneson *et al*. 2011; Abramyan and Stajich 2012; Liu and Stajich 2015; O’Hanlon *et al*. 2018; Wacker *et al*. 2023, 2024). Five lineages of *B. dendrobatidis* have been described to date (Farrer *et al*. 2011; Rosenblum *et al*. 2013; O’Hanlon *et al*. 2018), and reference genomes have been assembled for 2 of these lineages (*Bd*GPL and *Bd*Brazil), with recent efforts revealing pan-genomic variation in gene family counts (Yacoub and Stajich 2024). These efforts have been hampered by the fragmented, erroneous, and incomplete *B. dendrobatidis* JEL423 assembly, which includes long stretches of ambiguous positions and particularly poorly resolved telomeric regions. Here, we present and describe a substantially improved, near telomere-to-telomere genome assembly and gene annotation for *B. dendrobatidis* JEL423, which will be a crucial resource for future efforts to understand *B. dendrobatidis*' evolution to a pathogenic lifestyle.

Our new assembly is ∼800 kb longer (24.5 Mb) and comprises an extra ∼2.5% of repetitive sequence, with particular increases in unclassified and total interspersed repeats, LTR elements, DNA transposons, and retroelements, which have been recently shown to associate and potentially contribute to the expansion of pathogenicity genes in the batrachochytrids (Wacker *et al*. 2023, 2024). Mutator transposons are the most commonly identified DNA transposon in *dendrobatidis*, which are known to induce high mutation rates in other genomes and could drive genetic diversity and enhance pathogenicity (Dupeyron *et al*. 2019). Many Cacta elements were identified across the *B. dendrobatidis* genome, which are associated with chromosomal rearrangements (Badet *et al*. 2024), while copia retrotransposons identified can alter gene expression (Gao *et al*. 2008; Muszewska *et al*. 2011). Finally, Maverick elements were also common in *B. dendrobatidis* and are tied to viral function possibly contributing to genome defense and virulence factors (Kapitonov and Jurka 2006). Further work will be required to analyze and understand the distributions of repeats in the new assembly and particularly their associations with genes of interest including the M36 metalloproteases.

*B. dendrobatidis* JEL423 harbors an aneuploid genome, although it is unclear if this is found across multiple chytrid life stages, owing to the mixed cultures used for sequencing. We identified abundant heterozygosity, along with large stretches of homozygosity consistent with loss of heterozygosity events that may be the result of a hybrid origin for *Bd*GPL and/or parasex and mitotic recombination, supported further by hybrid genotypes being discovered (Farrer *et al*. 2011, 2021; Greenspan *et al*. 2018; O’Hanlon *et al*. 2018). We hypothesize that the base ploidy of *B. dendrobatidis* JEL423 is tetraploid, based on scaffolds with a decreased depth of coverage coinciding with bimodal allele frequencies peaking at 33 and 66% agreement of reference bases—which would suggest those are triploid. Indeed, other scaffolds increase in depth and have allele frequencies consistent with pentaploidy. Surprisingly, the specific counts of chromosomes and pattern of aneuploidy appear to be stable in culture and/or cryogenically frozen stocks over several years in *B. dendrobatidis* JEL423, based on the consistency of our new assembly and depth of coverage with 2018 Illumina reads (O’Hanlon *et al*. 2018), vs the old assembly and depth of coverage with the 2013 Illumina sequencing (Farrer *et al*. 2011, 2013). Further, potentially long-term evolutionary work will be required to check how long such aneuploidy can remain stable for.

Our new gene annotation revisited the counts of important gene families described previously in *B. dendrobatidis*, namely, M36 metalloproteases, CRNs, and CBM18s. We surprisingly identified fewer than half the number of M36 metalloprotease genes previously predicted, suggesting that many of those genes in the previous assembly of *B. dendrobatidis* were incorrectly identified duplications. The reduced number suggests that a smaller number of genes may be responsible for the destruction of skin and extracellular matrix in amphibians and/or that other genes and gene families have a larger role than previously thought, such as the aspartyl proteases and other secreted protein families encoded (Farrer *et al*. 2017). In contrast, CRNs remained abundant, albeit also slightly reduced in number, and remain enigmatic in function and evolutionary origin. CBM18s thought to bind and thereby limit exposure of fungal chitin (Liu and Stajich 2015; Farrer *et al*. 2017) were also found in greater numbers than previously recognized. The placement of one predicted CBM18 (g231) in our gene tree suggests that tyrosine-like CBM18s arose from lectin-like genes, albeit based on only preliminary and ad hoc gene classifications.

*B. dendrobatidis* remains a substantial challenge for ecosystem health and amphibian conservation efforts (Scheele *et al*. 2019). Our new genome assembly will be a useful tool for further investigation the genome evolution of the pathogenic chytrids, including deciphering genomic rearrangements, gene family changes (expansions and contractions), and pan-genomics. We also anticipate that the genome assembly will help inform the design of genetic transformation approaches to explore gene function and chytrid cell biology, such as those recently developed (Kalinka *et al*. 2024; Webb *et al*. 2024). High-quality genome assemblies such as our new *B. dendrobatidis* JEL423 assembly are urgently required in mycoinformatic studies, for which many species still lack a genome assembly at all, or have only draft assemblies, that can result in inaccurate descriptions of the genes and repeat content that may be pivotal for understanding their evolution and pathogenicity.

## Supplementary Material

jkae304_Supplementary_Data

## Data Availability

All ONT reads were uploaded to NCBI Sequence Read Achieve under project accession PRJNA1111108. All Hi-C reads were uploaded to NCBI Sequence Read Achieve under project accession PRJNA1111108. The annotated genome assembly for *B. dendrobatidis* JEL423 has been deposited to NCBI GenBank under project accession PRJNA1109278 and on FigShare (https://doi.org/10.6084/m9.figshare.27325284.v1). Supplemental material available at G3 online.
