# Supplementary material for: A near-complete telomere-to-telomere genome assembly for Batrachochytrium dendrobatidis GPL JEL423 reveals a larger CBM18 gene family and a smaller M36 metalloprotease gene family than previously recognized
Source: G3 (Bethesda). 2024 Dec 22;15(2):jkae304. doi: 10.1093/g3journal/jkae304 (PMC11797018; doi:10.1093/g3journal/jkae304)
Supplement: jkae304_Supplementary_Data [file jkae304_supplementary_data.zip › Supplemental_Material_Legends_G3-2024-405496.docx]

**Supplementary Information**

A near-complete telomere-to-telomere genome assembly for *Batrachochytrium dendrobatidis* GPL JEL423 reveals a larger CBM18 gene family and a smaller M36 metalloprotease gene family than previously recognised

**Figure S1.** Counts of all predicted proteases based on a BLASTp search of the MEROPS database, comparing the number found in the previous assembly (V2) with the new assembly (V3).

**Figure S2.** M36 metalloproteases were predicted in the *B. dendrobatidis* JEL423 (V3) gene annotation based on BLASTp search of the MEROPS database. Tblastn against the genome identified additional high scoring pairs over genes highlighted in red. All genes identified by MEROPS BLASTP or Tblastn were aligned using MUSCLE and a tree constructed using FastTree (branch lengths indicate the mean number of nucleotide substitutions per site).

**Figure S3.** Predicted crinkling and necrosis (CRN) genes in *B. dendrobatidis* JEL423 and three chytrid relatives based on a BLASTp search to the *P. infestans* T30-4 as previously done (Farrer et al. 2017b).

**Figure S4.** Predicted crinkling and necrosis (CRN) genes in *B. dendrobatidis* JEL423 based on a BLASTp search to the *P. infestans* T30-4 as previously done (Farrer et al. 2017b), revealed only 13 top HSPs after excluding splice variants (highlighted in red), along with 120 further genes identified from tblastn. The gene tree was constructed using FastTree. Branch lengths indicate the mean number of nucleotide substitutions per site.

**Table S1.** The number of telomere repeats identified on the 3; and 5’ ends of each scaffold of the new assembly.

**Table S2.** Transposable elements identified across each *B. dendrobatidis* JEL423 scaffold.
