# Supplementary figures and images for: A near-complete telomere-to-telomere genome assembly for Batrachochytrium dendrobatidis GPL JEL423 reveals a larger CBM18 gene family and a smaller M36 metalloprotease gene family than previously recognized
Source: G3 (Bethesda). 2024 Dec 22;15(2):jkae304. doi: 10.1093/g3journal/jkae304 (PMC11797018; doi:10.1093/g3journal/jkae304)

# Predicted proteases of Bd v2 and Bd v3

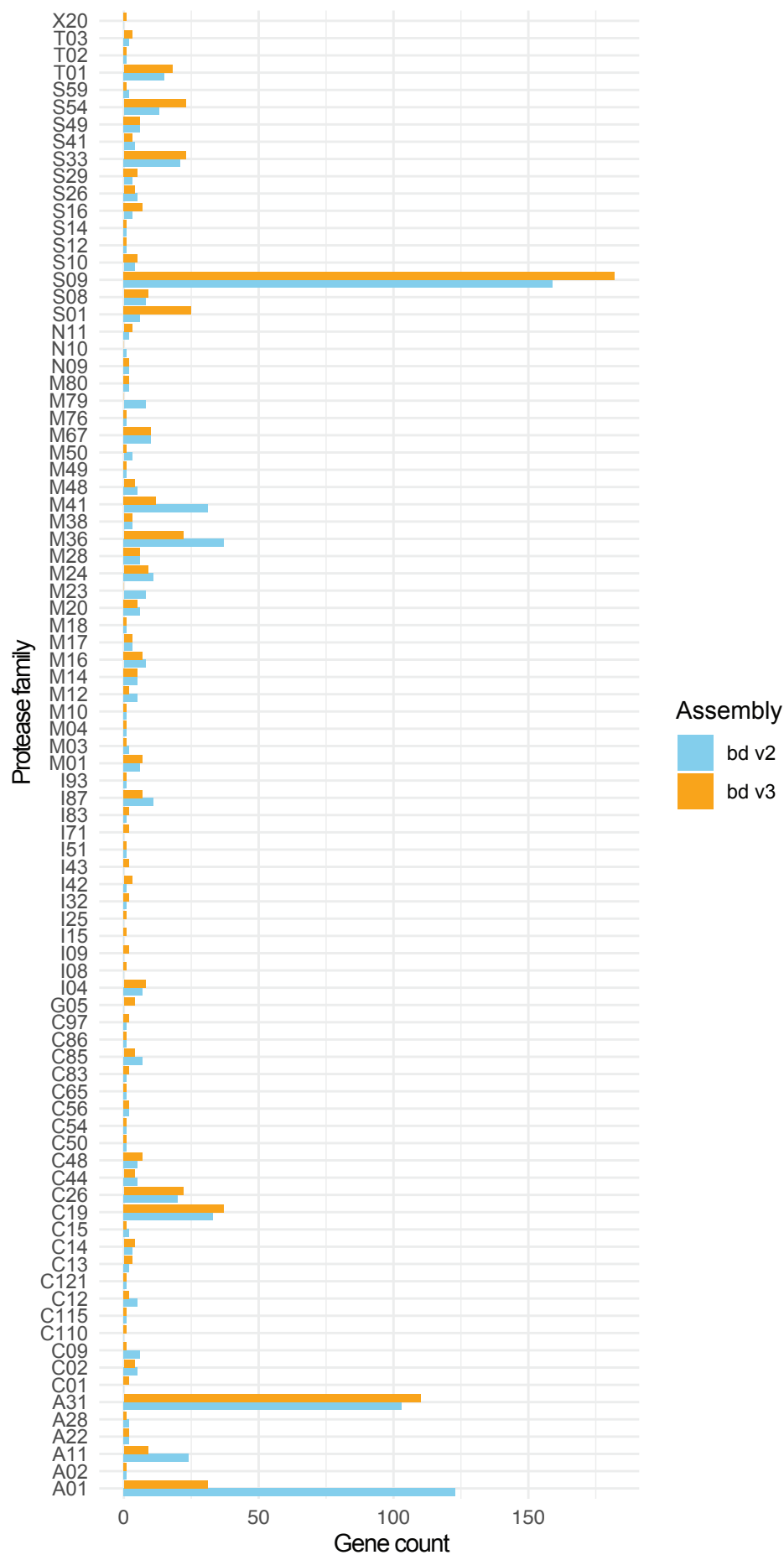

Supplement: jkae304_Supplementary_Data [file jkae304_supplementary_data.zip › Figure_S1_G3-2024-405496.pdf]

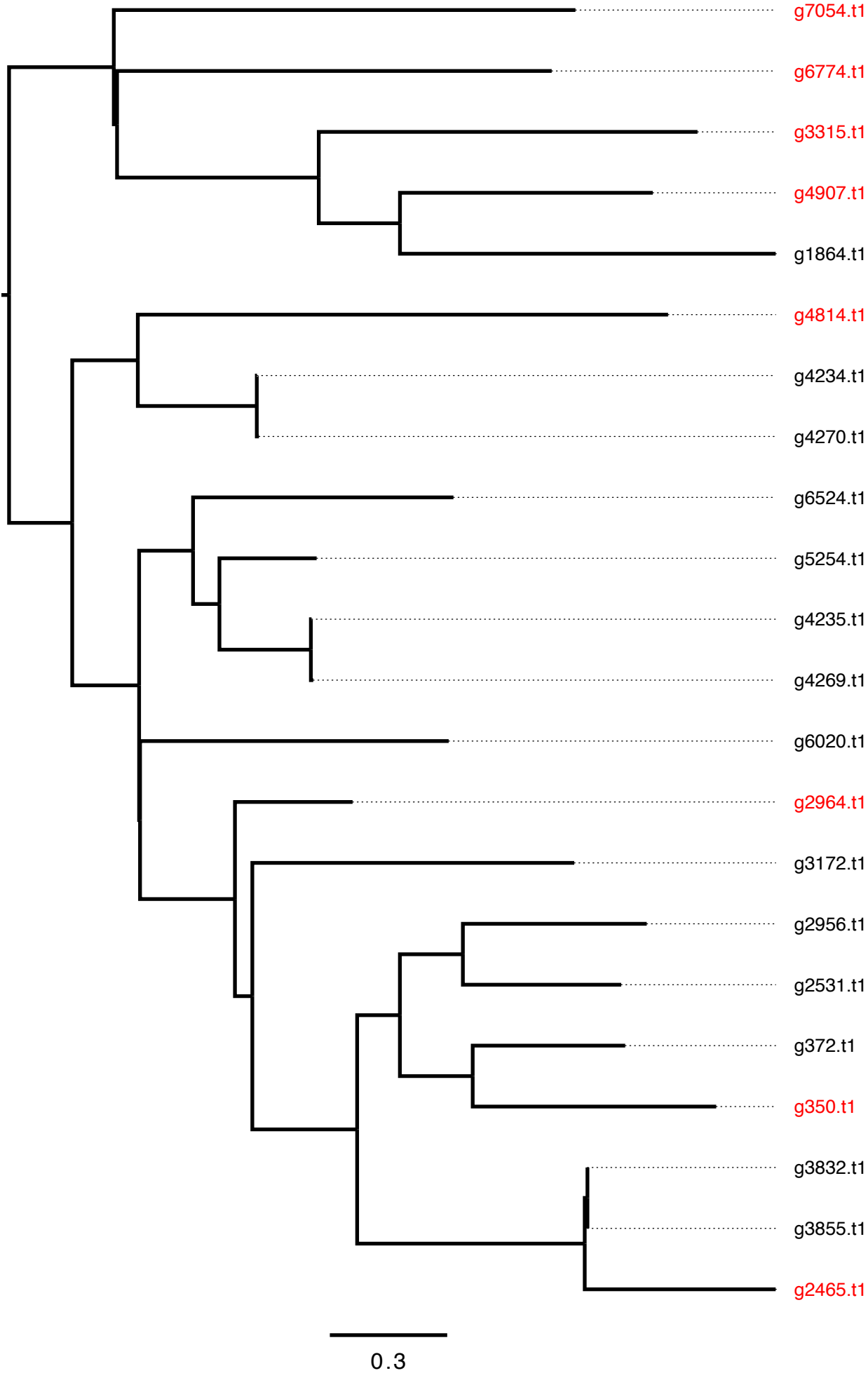

Supplement: jkae304_Supplementary_Data [file jkae304_supplementary_data.zip › Figure_S2_G3-2024-405496.pdf]

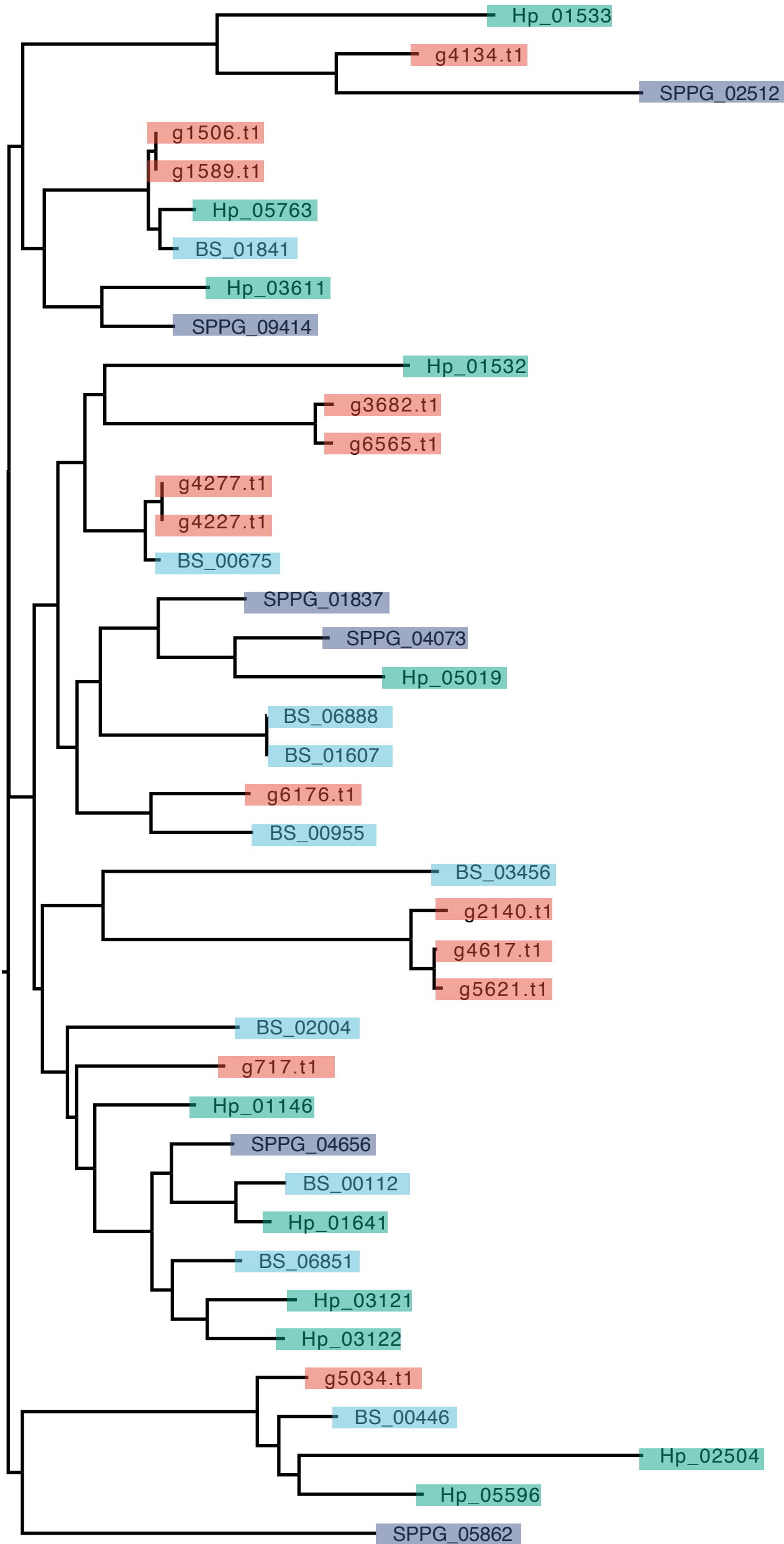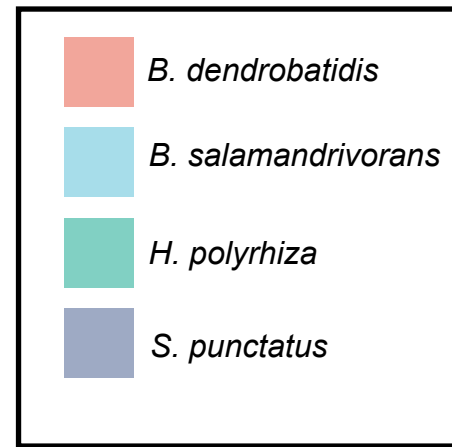

0.4

Supplement: jkae304_Supplementary_Data [file jkae304_supplementary_data.zip › Figure_S3_G3-2024-405496.pdf]

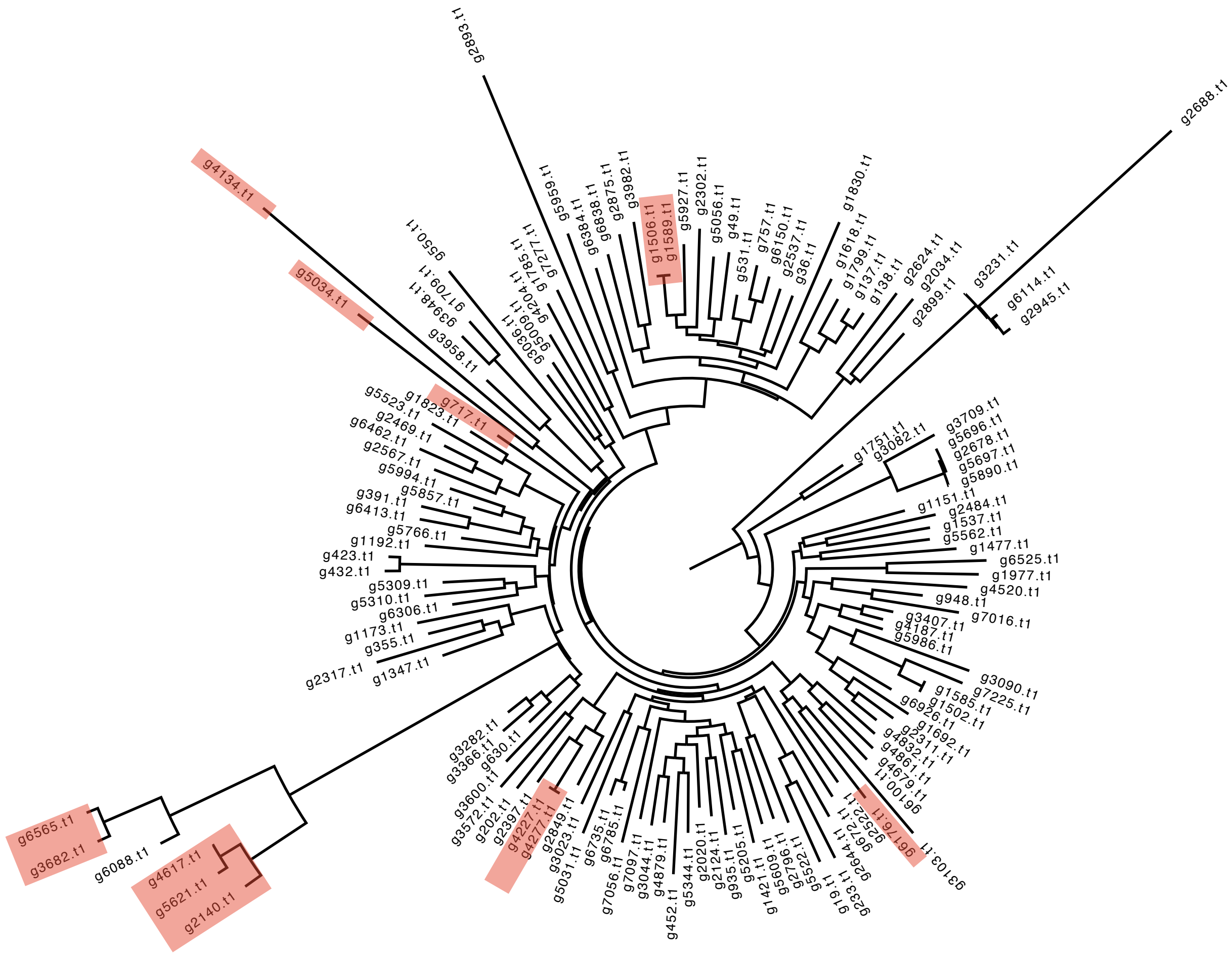

Supplement: jkae304_Supplementary_Data [file jkae304_supplementary_data.zip › Figure_S4_G3-2024-405496.pdf]
